# *Dictyostelium discoideum* Nucleoside Diphosphate Kinase C Plays a Negative Regulatory Role in Phagocytosis, Macropinocytosis and Exocytosis

**DOI:** 10.1371/journal.pone.0026024

**Published:** 2011-10-04

**Authors:** Sarah J. Annesley, Ruzica Bago, Maja Herak Bosnar, Vedrana Filic, Maja Marinović, Igor Weber, Anil Mehta, Paul R. Fisher

**Affiliations:** 1 Department of Microbology, La Trobe University, Victoria, Australia; 2 Division of Molecular Medicine, Rudjer Bošković Institute, Zagreb, Croatia; 3 Division of Molecular Biology, Rudjer Bošković Institute, Zagreb, Croatia; 4 Division of Medical Sciences, Ninewells Hospital and Medical School, University of Dundee, Dundee, United Kingdom; University of Birmingham, United Kingdom

## Abstract

Nucleoside diphosphate kinases (NDPKs) are ubiquitous phosphotransfer enzymes responsible for producing most of the nucleoside triphosphates except for ATP. This role is important for the synthesis of nucleic acids and proteins and the metabolism of sugars and lipids. Apart from this housekeeping role NDPKs have been shown to have many regulatory functions in diverse cellular processes including proliferation and endocytosis. Although the protein has been shown to have a positive regulatory role in clathrin- and dynamin-mediated micropinocytosis, its roles in macropinocytosis and phagocytosis have not been studied. The additional non-housekeeping roles of NDPK are often independent of enzyme activity but dependent on the expression level of the protein. In this study we altered the expression level of NDPK in the model eukaryotic organism *Dictyostelium discoideum* through antisense inhibition and overexpression. We demonstrate that NDPK levels affect growth, endocytosis and exocytosis. In particular we find that *Dictyostelium* NDPK negatively regulates endocytosis in contrast to the positive regulatory role identified in higher eukaryotes. This can be explained by the differences in types of endocytosis that have been studied in the different systems - phagocytosis and macropinocytosis in *Dictyostelium* compared with micropinocytosis in mammalian cells. This is the first report of a role for NDPK in regulating macropinocytosis and phagocytosis, the former being the major fluid phase uptake mechanism for macrophages, dendritic cells and other (non dendritic) cells exposed to growth factors.

## Introduction

Nucleoside diphosphate kinases (NDPK, nm23 or Nme family) are ubiquitous enzymes responsible for catalysing the phosphorylation of nucleoside diphosphates to nucleoside triphosphates via a labile, high energy phosphohistidine intermediate [1]. The nm23 family members are highly conserved from prokaryotes to eukaryotes and are responsible for producing most of the nucleoside triphosphates except for ATP. Apart from this housekeeping function, NDPKs have been shown to play numerous additional roles, the first of which to be discovered was as a suppressor of metastasis [2]. Subsequently, NDPK was found to participate in regulating proliferation [3]–[5], differentiation and development [6]–[10], apoptosis [11], [12], tumourigenesis [13], signal transduction [14], gene expression [15], [16] and vesicular trafficking [17]–[19].

Multiple types of endocytosis have been described in eukaryotic cells and involve distinct molecular mechanisms and cargos [20], [21]. Of these, phagocytosis and pinocytosis respectively facilitate the uptake of solid particles and fluid from the extracellular milieu, the latter being traditionally subdivided into macropinocytosis and micropinocytosis depending on the size of the resulting pinosome. Several distinct mechanisms of microscale endocytic processes (micropinocytosis) have been recognized and NDPK has been shown to play a positive regulatory role in some [22].

The best understood and most common type of endocytosis in cultured cells of higher eukaryotes is micropinocytosis mediated by clathrin coated vesicles. Macropinocytosis on the other hand, is favoured by specific cells such as macrophages, dendritic cells and cells which have been exposed to growth factors. Macropinocytosis involves the formation of large vesicles which result from membrane ruffles folding back on the plasma membrane [20], [21]. This process relies upon the polymerisation of actin and, like phagocytosis, does not involve clathrin. Although NDPK-B has been identified in the membranes of macrophages [23] and in the phagosomes of a murine macrophage cell line [24], the function of NDPK in phagocytosis and macropinocytosis has not been investigated previously. The model eukaryotic organism *Dictyostelium discoideum* is a tractable model for the study of phagocytosis and macropinocytosis [25] and hence was used in this work to study the role of NDPK in these processes.

*Dictyostelium* has a haploid genome which has been completely sequenced [26] and is amenable to a range of genetic manipulation techniques. It also has a unique life cycle in which starving amoebae differentiate, and aggregate chemotactically to form a multicellular organism (the so-called “standing finger/slug”) that subsequently undergoes further differentiation and morphogenesis to form a fruiting body. This life cycle, with its motile unicellular and multicellular stages and multiple cell types, provides an unparalleled variety of phenotypes for study. As such *Dictyostelium* has become a widely accepted model for the study of many cellular processes including signal transduction, chemotaxis [27]–[29], mitochondrial disease [30]–[32], movements of the cytoskeleton [33] as well as vesicle trafficking and endocytosis [25].

The *D*. *discoideum* genome includes three genes encoding proteins of the NDPK family. A mitochondrially located NDPK is encoded by *ndkM* (or *guk*), but accounts for only 2-3% of total NDPK activity [34]-[35]. Other eukaryotic organisms such as rats and humans also contain a mitochondrial NDPK with similar levels of activity [1]. Another gene (*DDB_G0292928*) encodes a protein that clearly belongs to the NDPK superfamily, but has not been studied. A third gene, *ndkC* (or *gip17*) encodes a cytosolic form of NDPK (NDPK-C), which represents most of the NDPK activity within these cells [36]. The hexameric form of NDPK-C was the first NPDK to be crystallized and its wild type and mutant forms provided a model for structure-function relationships in enzymes of the NDPK family [36]–[43]. However the biological roles of this protein in *Dictyostelium* have received scant attention. Here we redress this in a study of the biological functions of *Dictyostelium* NDPK. Overexpression and antisense inhibition studies presented here show that *Dictyostelium* NDPK-C (henceforth called NDPK for simplicity) plays roles in regulating growth, endocytosis and exocytosis. NDPK was found to inhibit phagocytosis and macropinocytosis which contrasts with its positive regulatory role in micropinocytosis. Mammalian NDPK is a metastasis suppressor and a link between macropinocytosis and metastasis in mammalian cells is suggested by the fact that a number of signalling proteins involved in metastasis are also implicated in macropinocytosis [20], [21]. Our observed biological functions for *Dictyostelium* NDPK are consistent with an ancient origin for some of its roles in growth and endocytic vesicle trafficking.

## Results

### NDPK subcellular localization

**Figure 1:**
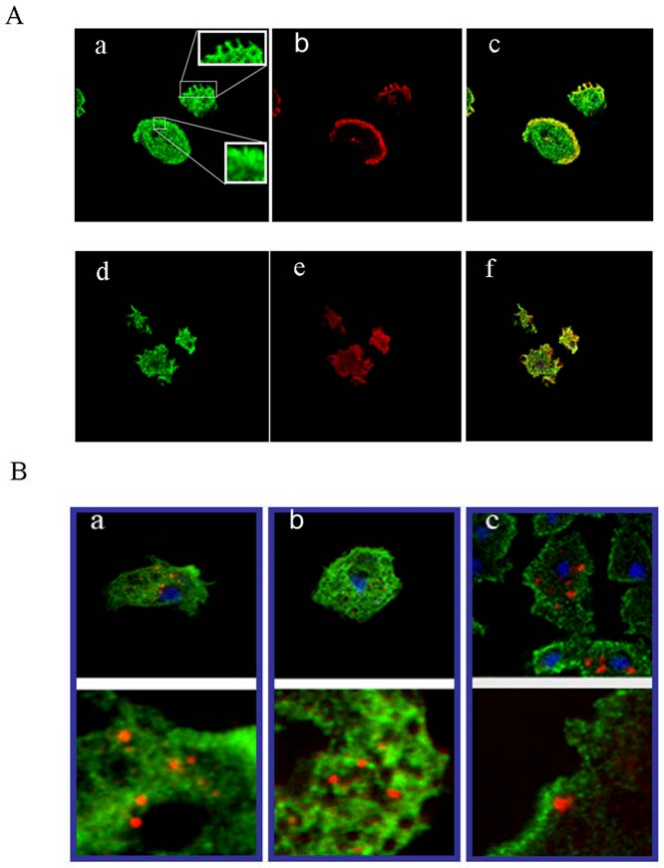
NDPK colocalises with actin and calmodulin, but not with macropinosomes. A. Wild type (AX2) *Dictyostelium* cells were immunostained with antibodies against NDPK, represented in green in panels a and d; actin, red in panel b; and calmodulin represented in red in panel e. The images were overlaid and association could be seen between NDPK and actin at the cell periphery in panel c and also between NDPK and calmodulin at the periphery and vacuoles in panel f. Insets in Panel A highlight enrichment of NDPK at the cell periphery and around vesicles and macropinosomic ruffles. B. Wild type (AX2) *Dictyostelium* cells in nutrient (HL-5) medium were pulsed with TRITC-dextran and chased for 0 minutes (panel a), 15 minutes (panel b) and 60 minutes (panel c). Cells were immunostained using antibodies against NDPK. TRITC-dextran fluorescence in macropinosomes is represented in red, NDPK immunofluorescence is shown in green and the nucleus is stained with DAPI, shown in blue. The lower part of each panel shows the NDPK localization and loaded macropinosomes at higher magnification.

Immunofluorescence microscopy of wild type AX2 *Dictyostelium* cells showed that *Dictyostelium* NDPK exhibits a punctate distribution in the cytoplasm with enrichment at the cell periphery and around vacuoles which may represent endocytic or exocytic vesicles (Fig. 1). In mammalian cells, NDPK also exhibits a punctate distribution through the cytoplasm and is more highly concentrated close to plasma and vesicular membranes [44]-[46]. This distribution of NDPK in *Dictyostelium* is reminiscent of the subcellular locations of actin and calmodulin, both of which are found distributed throughout the cytoplasm but enriched at the cell periphery (actin) or around vesicles (calmodulin) [47]–[48]. Our colocalisation experiments showed that NDPK was indeed enriched in regions containing higher concentrations of actin or calmodulin (Fig. 1A). However, coimmunoprecipitation experiments failed to support a stable biochemical interaction between NDPK and either actin or calmodulin (data not shown). Immunofluorescence studies of NDPK in animal cells similarly showed an association with the cytoskeleton [49], but no biochemical interaction could be identified [50].

Our results show that *Dictyostelium* NDPK exhibits a similar submembraneous localization to that of its mammalian counterparts and that this localization is independent of a stable interaction with either the actin cytoskeleton or calmodulin. These similarities suggested that, like its mammalian counterparts, *Dictyostelium* NDPK would participate in a variety of signalling pathways, including those that regulate endocytosis. As a first approach to this question we examined whether NDPK is located on endosomes during a pulse chase of TRITC-Dextran during its macropinocytic uptake from nutrient medium. TRITC-Dextran was pulsed for 5 minutes, removed from cells and chased for different time intervals 0 (a), 15 (b) and 60 (c) minutes. Each time point represents a different stage of endocytic vesicle trafficking and correspondingly different endosomal compartments. As the results show (Fig. 1B) NDPK was located close by, but not colocalized with TRITC-Dextran compartments at all chased time points. We conclude that NDPK is not a constitutive part of *Dictyostelium* macropinosomes.

### Creation of transformants with increased and decreased levels of NDPK expression

**Figure 2:**
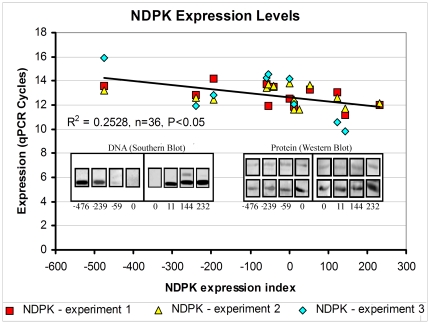
NDPK expression levels were altered in the transformants. Semiquantitative RT-PCR was performed three times for each of 12 transformants and the wild type strain and the threshold values obtained in each of the three experiments were plotted against the measured copy number data. Negative copy numbers refer to the antisense construct and positive copy numbers to the overexpression construct. These results show that NDPK expression levels were decreased in the antisense transformants and increased in the overexpression transformants giving an overall difference in expression of as much as 8-16 fold. The correlation was significant at P<0.05 (Pearson product moment correlation coefficient ρ). It accounts for about 25% of the variance in the qPCR threshold cycle number (R^2^ = 0.253), while the remainder reflects experimental error inherent in the qPCR and copy number measurements. Copy number was calculated by quantitative southern blotting. The inset on the left depicts the southern blots for several representative transformants and shows that the copy number varies between transformants. NDPK protein levels were also determined through western blotting and the results of several transformants are depicted in the inset labelled protein (western blotting). The upper panels in the western blots show the B-actin bands detected in the same blot as a loading control using a commercial antibody.

Although NDPK is not constitutively located on endosomes, its enrichment in submembraneous locations close to the cell periphery is consistent nonetheless with possible roles in endocytic vesicle trafficking. The biochemical structure-function relationships of the *Dictyostelium* enzyme have been well studied, but its biological roles have been less intensively investigated. To explore these, we created stable knock down and overexpression cell lines (strains) by transforming AX2 wild type cells with antisense inhibition or overexpression constructs. The integration of such constructs into random sites in the genome is accompanied by rolling circle replication of the plasmid, so that each transformant contains a different number of copies ranging typically from a few to several hundred [51]. The mere presence of multiple copies of integrated plasmid constructs has no observable phenotypic effects [32]. The variation in copy number permits the combination of overexpression and knock down strains with gene copy-number dependent dose response studies. This is a very powerful approach in which extraneous, unknown genetic changes that can arise stochastically during mutant isolation are readily excluded as sources of phenotypic changes. This is because such changes are random and not correlated with copy number. To determine how the variable gene dose affected NDPK expression, the copy numbers for the antisense and overexpression transformants were determined by quantitative Southern blotting and the NDPK mRNA levels were measured in semiquantitative qRT-PCR (Fig. 2). The results showed that the level of RNA was increased in the overexpression transformants and decreased in the antisense-inhibited transformants and that this was correlated with the copy number. The threshold cycle numbers in the qRT-PCR experiments ranged from 11 or 12 in the high copy number overexpression strains to about 15 in the highest copy number antisense strain. These correspond to differences in NDPK expression at the mRNA level of as much as 8- to 16-fold.

### NDPK has no effect on multicellular development

**Figure 3:**
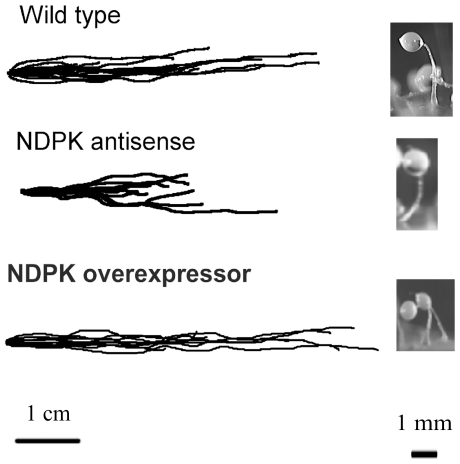
NDPK has no effect on phototaxis or fruiting body morphology. A:NDPK antisense-inhibited strains and NDPK overexpressing strains displayed accuracies of phototaxis in the wild type range. The light source is to the right of the figure. B:Fruiting body morphology was also unaffected in the transformants with their morphology resembling the wild type.

**Figure 4:**
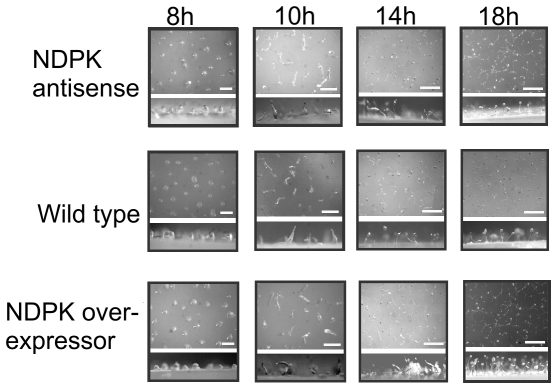
Multicellular development is unaffected by NDPK expression levels. The development of wild type, NDPK antisense and NDPK overexpressing cells was tracked over time and images recorded at the time points shown above the columns. Each panel includes an aerial view of the plate and a side view. All strains developed at a similar rate indicating that NDPK has no effect on the time course of multicellular development.

The *Dictyostelium* life cycle permits analysis of both the directional migration of the motile multicellular slug form and the gross morphology of the fruiting body (Fig. 3). We found no effects of either antisense inhibition or overexpression of NDPK on phototaxis or fruiting body morphology (Fig. 3) or developmental timing and morphogenesis throughout the developmental programme (Fig. 4).

### NDPK inhibits growth on bacterial lawns

**Figure 5:**
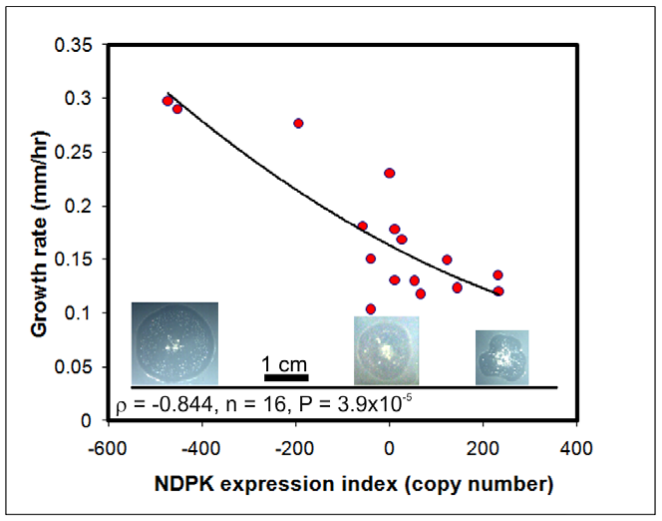
NDPK inhibits growth on bacterial lawns. The rate of expansion of wild type (0 copies – cells contain neither the antisense nor the overexpression construct) and NDPK transformant colonies (plaques) was measured in three independent experiments. The number of copies of the antisense (negative numbers) and overexpression (positive numbers) constructs within the genome were used as an NDPK expression index. Each point represents the mean of data obtained for a single transformant. Plaque expansion rates were negatively correlated with the NDPK expression index (copy number). The correlation was significant at P<0.01 (Pearson product-moment correlation coefficient ρ). Representative plaques (with scale bar) after 3 days incubation are shown for an antisense inhibition strain (left side - 473 copies), the wild type strain (middle - AX2) and an overexpression strain (right side - 232 copies).

In the single cell, vegetative phase of its life cycle, *Dictyostelium* engulfs bacteria as a food source and we observed effects on growth on bacterial lawns. Fig. 5 shows that a reduction in NDPK expression increases the plaque expansion rate on bacterial lawns, while overexpression results in a decrease in the rate of plaque growth on bacterial lawns. Contrary to these results, experiments by Sellam et. al. [52] did not reveal any growth defects in strains overexpressing NDPK. The larger effect in our results is observed with antisense inhibition and this may explain the discrepancy between these experiments and those conducted by Sellam et al [52] who did not study antisense-inhibited strains. Furthermore, multiple independent transformants were studied in our experiments and their phenotypes were correlated quantitatively with the copy numbers, allowing a more sensitive and accurate analysis of the phenotypic effects.

### NDPK inhibits phagocytosis but not amoeboid motility

**Figure 6:**
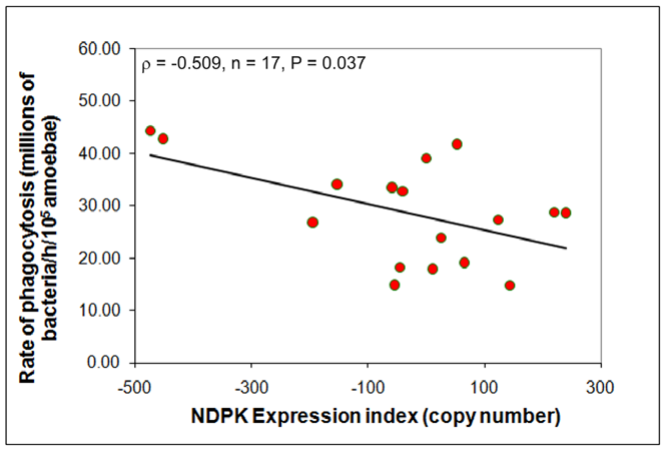
NDPK inhibits phagocytosis. The ability of the cells to ingest a fluorescent bacterium (DsRed-expressing *E.coli*) was assayed and showed that the phagocytic uptake of bacteria was negatively correlated with the NDPK expression index (defined as in Fig. 5). The correlation was significant at P<0.05 (Pearson product-moment correlation coefficient ρ). Each point is the mean for a single transformant from duplicate measurements made in each of five experiments.

Since elevated NDPK expression resulted in slower plaque expansion rates during growth on bacterial lawns (and *vice versa*), we wanted to determine if these phenotypes could be explained by changes in the ability of cells to ingest nutrients. To measure the rate of nutrient intake during growth on bacteria, phagocytosis experiments were conducted. As seen in Fig. 6, increasing the levels of NDPK expression resulted in decreased rates of phagocytosis, thereby providing an explanation for the reduced rate of plaque expansion on bacterial lawns.

**Figure 7:**
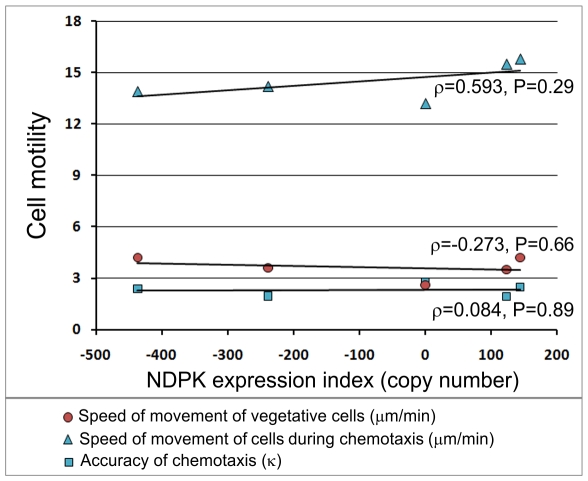
Cell motility is unaffected by the level of NDPK expression. Random motility by vegetative cells and chemotactic motility of aggregation-competent cells of the wild type strain (AX2) as well as two antisense and two overexpression transformants were assayed in three independent experiments. Each point represents the overall mean velocity of movement or the accuracy of chemotaxis (κ) from all three experiments combined. κ is the concentration parameter of the von Mises distribution for directional data [87] and ranges from zero in the case of no orientation to infinity in the case of perfect orientation (all directions identical). Tables S1 and S2 show, in pairwise statistical comparisons, that the slight motility differences amongst the strains were real (statistically significant) but as shown in the Figure they were not correlated with NDPK expression.

The ability of the amoebic form of *Dictyostelium* to grow on bacterial lawns is controlled not only by their ability to ingest bacteria via phagocytosis but also by their motility as the cells need to migrate into the bacterial lawn in order to consume the bacteria. Therefore, we measured random motility by vegetative cells and chemotactic motility by starved, aggregation competent cells of NDPK antisense and overexpression transformants on glass coverslips (Fig. 7, Tables S1 and S2). We found no effect of NDPK expression levels on motility under these conditions, suggesting that the reduced growth on bacterial lawns is indeed caused by decreased rates of phagocytosis. Since motility is unaffected, the inhibition of phagocytosis is unlikely to be mediated by general impairment of actin assembly or pseudopod extension.

### NDPK promotes growth in liquid, but inhibits macropinocytic vesicle uptake and exocytosis, increasing vesicle residence time

**Figure 8:**
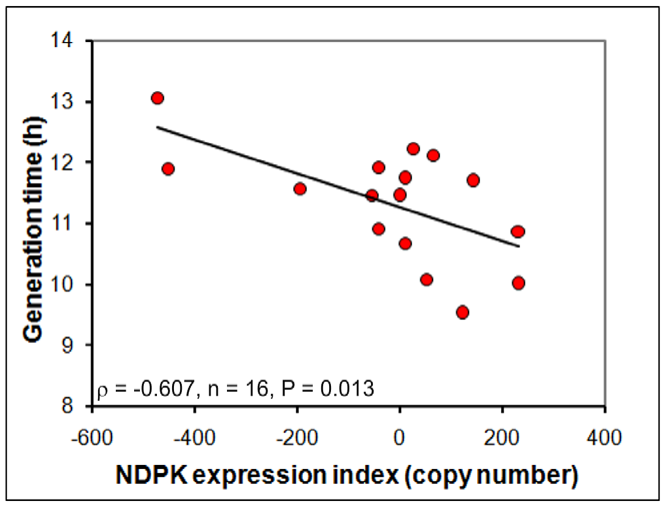
NDPK stimulates growth in liquid medium. The generation time for wild type and NDPK transformants was measured in three independent experiments and the mean for each transformant was plotted against the NDPK expression index (defined as in Fig. 5). The generation times decreased as the level of NDPK expression increased. The correlation was significant at P<0.05 (Pearson product-moment correlation coefficient ρ).

**Figure 9:**
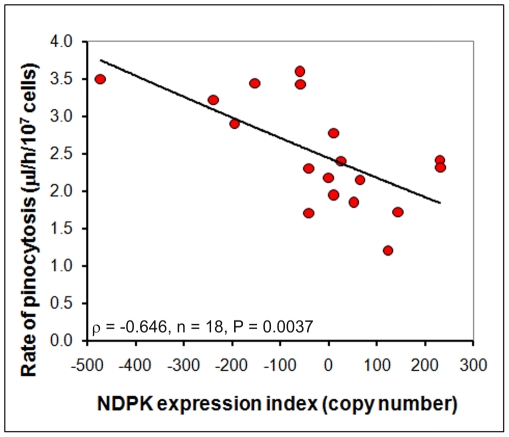
NDPK inhibits macropinocytosis. The ability of axenically growing cells to uptake FITC-dextran was measured and plotted against the NDPK expression index (defined as in Fig. 5). Macropinocytosis rates declined as NDPK expression levels increased. The correlation was significant at P<0.01 (Pearson product-moment correlation coefficient ρ). Duplicate measurements were made in each of three experiments for each transformant and the overall mean from all three experiments was plotted.

**Figure 10:**
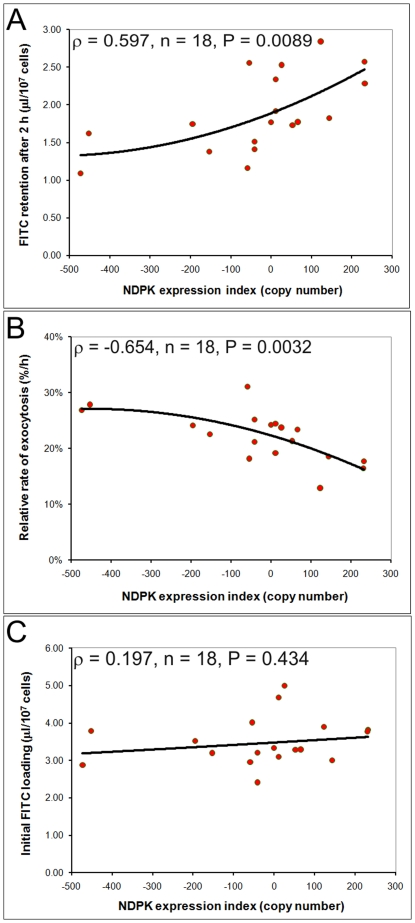
NDPK inhibits exocytosis. Duplicate measurements of the rate of exocytosis of FITC-Dextran were made in three independent experiments and the mean for each transformant plotted against the NDPK expression index (defined as in Fig. 5). **A.** Two hours after washing, the amount of FITC retained in the cells increased with the level of NDPK expression. The amount of FITC retained in the cells is expressed in terms of the equivalent volume of FITC-containing medium at the time of pinocytic uptake. **B.** The rate of exocytosis decreased as the level of NDPK expression increased. **C.** The steady state loading of FITC-dextran-containing medium was unaffected by NDPK expression levels. The correlation in both A and B was significant at P<0.01, while the correlation in C was not significant at P>0.1 (Pearson product-moment correlation coefficient ρ). Lines of best fit in A and B were plotted using a 2^nd^ order polynomial function which provided a visually better fit to the data than the best fitting linear function. A linear function was fitted in C.

Laboratory strains of *Dictyostelium* are able to grow axenically (i.e. without a bacterial food source) in liquid medium, a mode of growth that is supported by macropinocytic uptake of nutrients from the broth. Whereas elevation of NDPK expression levels inhibited growth on bacterial lawns, high NDPK copy number produced an increase in the rate of growth (i.e. decreased generation time) in axenic (liquid) culture (Fig. 8). Unexpectedly, we found that this higher growth rate could not be explained by a corresponding increase in the rate of pinocytic nutrient uptake. Instead, macropinocytosis, like phagocytosis, was inhibited by increased NDPK expression levels (Fig. 9). To investigate this apparent paradox, we determined the rate at which the contents of endocytic vesicles were expelled by exocytosis. The results in Fig. 10 show that 2 hours after removal of FITC from the extracellular medium, significantly higher levels were retained by the cells when NDPK expression levels were higher (Fig 10A). This increase in the residence time within the cell of material taken up by macropinocytosis resulted from a reduction in exocytic rates (Fig 10B), while the steady state mass of endocytosed material was not significantly affected (Fig 10C). These results suggest a possible explanation for the paradoxically faster growth in liquid medium as NDPK expression levels increase - the longer residence time of macropinocytic vesicles within the cell at higher NDPK levels may allow more complete digestion and uptake of available nutrients from within the vesicles.

## Discussion

We have investigated the biological roles played by NDPK in *Dictyostelium* by altering its expression levels. Expression was knocked down by antisense inhibition or increased by overexpression from a constitutively active promoter. NDPK is normally expressed at high levels in *Dictyostelium* vegetative cells and down-regulated drastically during the first 6 hours of starvation-induced development [35]. This is followed by a slight increase to levels which are stable for the rest of development.

The development of *Dictyostelium* as a multicellular organism depends upon the differentiation of two major cell types, spore and stalk cells, and their proper distribution in the fruiting body by a series of complex morphogenetic movements. We found that developmental timing, multicellular morphogenesis and final fruiting body morphology were unaffected by altered NDPK expression levels. This is consistent with the normal expression patterns for NDPK and suggests that the protein plays its major roles in *Dictyostelium* during vegetative growth rather than later in development. However we cannot rule out subtle developmental defects which do not cause obvious alterations in morphogenesis. It is also possible that NDPK is required for normal fruiting body morphology but that in our experiments, the levels were not altered sufficiently to produce an obvious phenotype. In other organisms where developmental defects were observed, the gene had been knocked out [53]–[54].

Amongst the most intensively studied roles of NDPK are its functions in tumourigenesis and metastasis. The former involves uncontrolled proliferation of tumour cells and the latter involves their migration to secondary sites. Throughout this process, tumour cells are dependent on their ability to ingest nutrients from the extracellular milieu surrounding them. In this work we have studied the roles of NDPK in *Dictyostelium* proliferation, motility and nutrient uptake.

An increase in NDPK expression is generally associated with increased cell growth as inferred from data in cultured mammalian cells in which knock down of NDPK resulted in cell growth suppression [3], [55]. In *Dictyostelium* we observed a similar result, with decreased NDPK expression resulting in decreased axenic growth and *vice versa*. Growth on bacterial lawns did not display this association, but this can be explained by the effects on phagocytosis, a form of nutrient uptake that did not pertain in the mammalian cell experiments.

Although the difference between growth on plates and axenic growth was unexpected, such differences have been noted before. Alteration of the expression levels of NDPK often exerts different effects depending on the conditions. This plasticity of NDPK action can occur even within the one kind of cell. For example, in LMF4 clones, depending on the environmental conditions, NDPK either potentiates cell growth or inhibits it to promote cellular differentiation and development [56]. Our experiments similarly showed that NDPK affects *Dictyostelium* growth in opposing ways in different cellular environments.

NDPK was first identified as a metastatic suppressor in mouse melanoma cell lines [2] and since then has also been shown in numerous (but not all [57]) metastatic cell lines to be down regulated (review in [13]). Motility is an important aspect of metastasis allowing the cells to migrate to secondary sites. *In vitro* experiments on cancer cell lines show that NDPK reduces cell motility and that this is accompanied by reduced cell invasion. It is known that the role of NDPK in cell lines is not related to its enzymatic activity but to its expression levels [58]. In our experiments we found no effect of NDPK expression levels on cell motility when we measured the motility of axenically grown cells on glass. However, we cannot exclude possible effects of NDPK expression levels on the movement by which growing amoebae invade a bacterial lawn on the surface of agar plates. These conditions may be more comparable to the emigration from primary tumours and invasion of other tissues by metastatic cells.

Our finding that NDPK reduces both pinocytosis and exocytosis could be related to its endocytic role in animal cells. For example mammalian NDPK-A can interact with ARF6-GTP a protein which is involved in the disassembly of adherens junctions [19]. ARF6-GTP interacts with and recruits NDPK-A to the cell membrane which aids in the process of endocytosis. Additionally NDPK-B has been shown to directly interact with ICAP-1α (Integrin Cytoplasmic Domain-Associated Protein), and forms a complex with ICAP-1α, β-integrin and Rac1 on the cell membrane during early adhesion [59], [12]. NDPK has also been shown to play a role in intracellular vesicle trafficking. For example, NDPK-B interacts with COPII a complex which is essential for mediating vesicle formation at the ER and for ER to Golgi trafficking [60]. In *Drosophila* NDPK regulates dynamin-dependent internalisation processes such as synaptic vesicle endocytosis and vesicle-dependent turnover of receptors [18]. NDPK has also been shown to interact with cytoskeletal modulators which are important for endocytosis including an indirect association with Tiam1 [61].

During the final stage of *Dictyostelium* development, prespore secretory vesicles (PSV) are exocytosed from prespore cells to form the outermost layer of spore cell wall. The proteins in these vesicles contain not only the coat proteins but also regulatory proteins such as NDPK [62]. This localization of NDPK to PSV and the results presented here suggest that NDPK may be involved in regulating exocytosis and endocytosis in *Dictyostelium* as in animal cells.

However the *direction* of NDPK-mediated regulation of endocytosis and exocytosis observed here, contrasts with what has been observed in some other systems. For example, in *Drosophila*, NDPK rescued the endocytic and synaptic recycling defects displayed by a dynamin null mutant [18]. This suggests that metazoan NDPK plays a positive regulatory role in endocytosis compared with the negative regulatory role observed in *Dictyostelium*.

How can these apparently contradictory observations be explained? The answer may lie in differences in the types of endocytosis predominantly employed by *Dictyostelium* and cultured cells of higher eukaryotes. Endocytosis and exocytosis are important to all eukaryotic cells for the uptake of nutrients and expulsion of waste, recycling of receptors etc. However, endocytosis can be performed by several different mechanisms including macropinocytosis and clathrin- or dynamin-mediated micropinocytosis. Specific cells favour a particular method of fluid phase uptake - either macropinocytosis or micropinocytosis. When one method is favoured, the other is down regulated and *vice versa*. While macropinocytosis accounts for the majority of fluid phase uptake in *Dictyostelium* [63], in cultured cells of higher eukaryotes fluid phase uptake mostly occurs through micropinocytosis (reviewed by [20], [21]). Macropinocytosis predominates only in select types of cells, including macrophages and dendritic cells, and can also be induced in (non-dendritic) cells by exposure to growth factors [64]–[68].

Although NDPK promotes clathrin-dependent and dynamin-dependent micropinocytosis (in animal cells), our results show that it inhibits macropinocytosis (in *Dictyostelium*). This suggests that NDPK may be a regulator of the type of endocytosis used and is consistent with the roles of other pinocytic regulators. For example in mammalian cells NDPK-A inhibits Rac1 thereby causing increased micropinocytosis [61], yet in *Dictyostelium* Rac1 overexpression results in increased macropinocytosis [69]. Thus Rac1 appears to be involved in inhibiting micropinocytosis and promoting macropinocytosis. There is evidence to suggest that the formation of membrane ruffles and macropinocytosis is related to oncogenic transformation and the metastatic potential of tumour cells. The normal and oncogenic forms of both Ras [70]–[71] and Src [72] stimulate increased rates of macropinocytosis and, in the latter case at least, this can be inhibited by expression of the metastatic suppressor drs. Metastasis suppressors thus seem to reduce the macropinocytic ability of cells and our results with NDPK are consistent with this. The mechanisms underlying the link between metastasis and macropinocytosis are largely unknown, but some of the same signalling molecules appear to be involved e.g. Ras proteins and PI3 kinases [20], [21], [73]–[76]. There are many Ras proteins in *Dictyostelium* and some are known to be involved in macropinocytosis [77]–[79]. Similarly double knockout of two of the three PI3 kinases in *Dictyostelium* produces defects in macropinocytosis [80]–[81]. Together these results suggest that the study of macropinocytosis in the genetically tractable model *Dictyostelium discoideum* may shed light not only on the regulation of endocytic pathways, but also indirectly on the complex process of metastasis in mammalian cells.

## Materials and Methods

### *Dictyostelium* strains and culture conditions

*Dictyostelium* wild type strain, AX2 [82], and the derived transformants were either grown axenically in HL-5 medium [82] which for transformed cell lines, contained 15 µg/ml G418 (Promega, Annandale, Australia) in shaken suspension (150 rpm) at 21°C or on SM agar plates with *Klebsiella aerogenes* as a food source [83]. The transformants used included NDPK antisense inhibited strains (containing construct pPROF500) and NDPK overexpressing strains (containing construct pPROF520).

### Plasmid Construction

Full length *ndkC* was amplified from genomic AX2 DNA using primers NDPKF (ATCTGAGAATTCATCGATATGTCCACAAATAAAGTAAA) and NDPKR (ATCTGAGAATTCCTCGAGTTATTCGTATAAATTTGGGT).

The PCR product was cloned into the *Eco*RI site of an *E. coli* vector pUC18 and named pPROF519. The gene was subcloned into the *Dictyostelium* expression vector pA15GFP in the sense orientation using the restriction enzymes *Xho*I and *Cla*I and named pPROF520. This construct was used for overexpression of the NDPK gene. The gene was also subcloned from pPROF519 into the *Eco*RI site of the *Dictyostelium* vector pDNeo2 in the antisense orientation to create the construct pPROF500 which was used for antisense inhibition experiments.

### Transformation

AX2 cells were transformed with 20 µg of non-linearised construct DNA (pPROF500 or pPROF520 for creation of NDPK antisense inhibition or NDPK overexpression strains) via the calcium phosphate DNA precipitation method [84] Following selection on *Micrococcus luteus* lawns grown on SM agar plates containing 15 µg/ml G418 [85] transformants were subcultured and maintained on *Klebsiella* lawns and axenically in HL-5.

### Growth Assays

#### Growth on solid media

A scraping of amoebae was subcultured using a flat edged toothpick to the centre of a Normal Agar (20 g/l agar [Oxoid, Basingstoke, Hampshire, England], 1 g/l Bacto peptone [Difco, Detroit, MI], 1.1 g/l anhydrous glucose, 1.9972 g/l KH_2_PO_4_ and 0.356 g/l Na_2_HPO_4_.2H_2_O, pH 6.0) plate containing a lawn of *E.coli* B2. The diameter of the plaque was measured at 8–12 hour intervals for an average of 100 hours. The recorded values were analysed by linear regression by using the “R” environment for statistical computing and graphics (http://www.R-project.org) to determine the plaque expansion rate.

#### Growth in liquid media

Growth in liquid medium was performed as previously described [86]. *Dictyostelium* wild type and transformant cell lines were grown axenically to exponential phase in HL-5 with no antibiotics. Cells at an initial cell density of 1×10^4^ cells/ml were subcultured to fresh HL-5 medium (no antibiotics) and the cell densities were determined using a haemocytometer at 8–12 hour intervals over a period of 100 hours. The cell densities were then analysed by log-linear regression using the R programming environment computer software to determine the generation time from the exponential portion of the growth curve.

### Endocytosis Assays

All *Dictyostelium* strains (wild type AX2 and transformants) were grown in HL-5 medium with no antibiotics to exponential phase prior to use in the following experiments.

#### Phagocytosis Assay

Bacterial uptake by *Dictyostelium* strains was determined as previously [32] by using as prey an *E.coli* strain expressing a fluorescent protein DsRed [87]. *Dictyostelium* amoebae were harvested, washed, resuspended at 1.3-23×10^6^ cells/ml and starved in Sorenson's buffer at 21°C for 30 min with shaking at 150 r.p.m. For the assay, 1 ml of the *E. coli* suspension was added to 1 ml of starving *Dictyostelium* cells. At each time point in the assay, 0.5 ml amoebae were washed free of uningested bacteria by differential centrifugation in the presence of 5 mM sodium azide and their fluorescence was measured in a Turner Biosystems Modulus fluorometer using a specially constructed module designed for DsRed (530 nm excitation and 580 nm emission). Measurements were performed in duplicate at each time point. The hourly rate of consumption of bacteria by a single amoeba was calculated from the increase in fluorescence over 30 min, the fluorescence signal per million bacteria and the amoebal density.

#### Pinocytosis Assay

Pinocytosis assays [88] were performed using fluorescein isothiocyanate (FITC-dextran, Sigma-Aldrich, average mol. mass 70 kDa; working concentration, 2 mg/ml in HL-5 growth medium) as previously described [32]. Axenically growing cells were harvested, resuspended in HL-5 at 2.5–25×10^6^ cells/ml, and shaken at 150 rpm for 20 min at 21°C. FITC-dextran was added to the cells, and at each time point 200 µl aliquots were transferred to 3 ml of ice-cold phosphate buffer (2 mM Na_2_HPO_4_. 2H2O and 15 mM KH_2_PO_4_, pH 6.0). The cells were harvested, washed twice with ice-cold phosphate buffer, and lysed by addition of 2 ml of 0.25% (vol/vol) Triton X-100 in 100 mM Na_2_HPO_4_, pH 9.2. The fluorescence of the lysate was measured in duplicate at each time point in a Modulus fluorometer (Turner BioSystems) using the Green Module. The hourly rate of uptake of medium was calculated from the cell density, the increase in fluorescence over 60 or 70 min, and a separate calibration curve relating the fluorescence signal to the volume of fluorescent medium.

#### Exocytosis Assay

The assay was performed as in the pinocytosis assay [32] except the cells were incubated in FITC-Dextran for 90 minutes to achieve complete steady-state FITC-Dextran loading of the cells. The cells were pelleted by centrifugation (2,500 x g/2 minutes) and resuspended in HL-5 containing no FITC. Duplicate 200 µl aliquots were removed (T0), the cells were incubated for 2 hours and a duplicate 200μl aliquots were again removed (T2). The aliquots were added to tubes containing 3 ml of Sorensen's buffer, washed counted, lysed and the raw fluorescence measured as in the pinocytosis assay.

### Western Blot

For western blotting, 1×10^7^ cells were lysed in 200 µl of lysis buffer (50 mM Tris-HCl, 150 mM NaCl and 0.02% Triton-X100) centrifuged and the supernatant was transferred to a new tube. A 15 µl aliquot of protein extract in 4 x loading buffer (62.5 mM Tris-HCl, pH 6.8, 25% glycerol, 2% SDS, 0,01% bromophenol blue) was boiled for 5 minutes, subjected to 10% SDS-PAGE, transferred to PVDF membrane (Bio-Rad) and incubated in primary rabbit antiNdkC (affinity purified, a kind gift from Dr. M.L. Lacombe, 1∶1000), or mouse antiactin (Santa Cruz Biotech 1∶1000) antibodies followed by incubation in secondary antirabbit (Santa Cruz Biotech.) or antimouse (Amersham) antibodies, both 1∶2,500. Proteins were visualised using the chemiluminescence ECL system (Amersham) and the STORM image analyser (Amersham).

### Immunofluorescence

Exponentially growing AX2 cells were incubated for 1 hour in HL-5 followed by incubation for 30 minutes in LoFlo HL-5. Cells were fixed in 4% formaldehyde, washed with PBS, permeabilised ice-cold methanol, blocked in blocking buffer (1% BSA, 1% fish gelatin, 0.05% Tween 20 in PBS), and labelled overnight at 4°C with primary rabbit antiNdkC and mouse antiactin (Santa Cruz Biotechnology) antibodies in blocking buffer. Next day the cells were incubated in secondary antimouse Alexa 594 and antirabbit Alexa 488 antibodies for 1 hour at room temperature. Finally, the cells were mounted in mounting medium (Dako) containing 4′,6-diamino-2-phenyl-indol (DAPI, Sigma).

### Confocal microscopy

All fluorescent images were obtained using a Leica TCS SP2 AOBS confocal microscope equipped with an HCX PL APO λ-Blue 63x/1.4 objective (Leica Microsystems). Alexa-594 was excited using a 594 nm laser line, FITC by a 488 nm laser line, and DAPI by a 405 nm laser line. Optical sections were recorded using a 103, 77 nm×103, 77 nm×850 nm voxel size. Images were processed and analyzed in AdobePhotoshop 7.0.

### TRITC-Dextran pulse chase

AX2 cells were prepared as for the immunofluorescence protocol. Cells were pulsed with 2 mg/ml TRITC-Dextran in Lo-Flo for 5 minutes, rinsed with PBS, and chased in Lo-Flo for 0, 15 and 60 minutes. Cells were fixed in 4% paraformaldehyde, permeabilized in 0.2% Triton X-100 and incubated with antiNdkC antibody, as previously described.

### Calculation of construct copy numbers by quantitative Southern blotting

Genomic DNA was extracted from cells using DNAzol (Molecular Research Center, CI, Ohio) and the instructions provided by the manufacturer. Half of the genomic DNA sample was used in a Southern blot after digestion with appropriate enzymes to release the cloned gene. The signals were detected using a fluorescently labelled probe for NDPK in combination with the enhanced chemiluminescence system (ECL, Amersham Biosciences). The other half of the sample was used undigested in Vistra Green (Amersham Biosciences)-stained gels to determine loading amounts. Standard curves for the Southern blot were created by probing known quantities of the gene released from the plasmid construct in question. Standard curves for the loading control gel were created using purified DNA of the linearized plasmid construct.

### *q*RT-PCR

Expression of NDPK mRNA was quantitated by real time RT-PCR by using the iQ5 real-time PCR detection system. The expression of filamin was also quantitated and used as a loading control. cDNA synthesis was carried out at 42°C for one hour using Moloney murine leukemia virus reverse transcriptase (Promega, Madison, WI) (200 U) and the 3′ primer NDPK432R (ccgatcattaaacgggc) or FIL1688R(ccatctaaacctggacc) according to manufacturers instructions. 10 µl of this cDNA was used in a 50 µl reaction mixture containing 1x SYBR Green Supermix (Bio-Rad, Hercules, CA) (100 mM KCl, 40 mM Tris-HCl, pH 8.4, 0.4 mM of each dNTP, iTaq polymerase (50 U/ml), 6 mM MgCl_2_, SYBR Green I, 20 mM fluorescein, and stabilisers) and gene specific primers (250 nM) FIL1588F (ccctcaatgatgaagcc) FIL1688R and NDPK330F (gaccattcttcggtgg) and NDPK432R. The PCR cycling used 30 seconds of denaturation at 95°C, annealing at 55°C for 30 seconds and elongation for 30 seconds at 72°C. Expression levels were normalized against the filamin levels to adjust for loading and then measured relative to AX2 control cells.

### Phototaxis Assay

Qualitative phototaxis tests were performed by transferring a toothpick scraping of amoebae from a colony growing on a *K. aerogenes* lawn to the centre of charcoal agar plates (5% activated charcoal, 1.0% agar). Phototaxis was scored after a 48 hour incubation at 21°C with a lateral light source. Slug trails were transferred to PVC discs, stained with Coomassie Blue and digitized. The orientation of the slug migration was analysed using directional statistics [89].

### Development of cells on KK2 agar plates

Exponentially growing *Dictyostelium* cells were harvested by centrifugation at 2,000 rpm for 2 min and washed thrice in cold sterile Development Buffer (DB) (5 mM Na_2_HPO_4_, 5 mM KH_2_PO_4_, 1 mM CaCl_2_, 2 mM MgCl_2_, pH 6.5). The cell pellets were resuspended in DB and plated onto KK2 agar plates (16 mM KH_2_PO_4_, 4 mM K_2_HPO_4_, 1.5% agar) at a density of 2.5×10^6^ cells/cm^2^. The plates were wrapped in wet paper towel and plastic wrap and incubated at 21°C for the desired period of time.

### Cell motility assays

For random motility assays, cells were grown on plastic cell-culture dishes under submerged conditions at subconfluent densities, and were transferred into antibiotic-free medium 18–24 hours prior to measurements. Cells were replated at a low density onto glass coverslips, so that there were not more than 25 cells in the 1260×912 µm field of view, and allowed at least two hours to settle. Images of moving cells were recorded using a CCD camera fitted onto a Zeiss Axiovert 135 inverted microscope equipped with a long-working-distance condenser (INA 0.55) and a 5× objective (NA 0.15). Illumination was adjusted equivalently to a dark-field optical configuration, so that the cells appear bright against the dark background, thereby facilitating the subsequent image processing. The interval between recorded images was 30 seconds. Individual cell tracks were identified by the ParticleTracker plugin module for ImageJ image processing software [90]. Speeds for each cell were calculated using a dedicated routine written in Mathematica v.7©.

Chemotaxis experiments were performed in a Dunn chamber [91]. The inner well of the chamber was filled with the phosphate buffer and the outer well with a 100 nM cAMP solution. Cells were allowed to develop for 6 to 7 hours on plastic cell-culture dishes in phosphate buffer until aggregation commenced, and were subsequently transferred into the chamber at low density. Measurements started 30 minutes after the transfer, and lasted for two hours. The interval between recorded images was 60 seconds. Cell displacements were calculated for each time interval and results pooled from three experiments for each strain.

### Statistical analysis

Correlation analysis used the Pearson product moment correlation coefficient (ρ) and regression lines were fitted by the least squares method to linear or 2^nd^ order polynomial functions, whichever provided a visibly better fit to the data. Nonparametric analysis using the Kendall (τ) and the Spearman (ρ) rank correlation coefficients produced very similar significance probabilities and the same conclusions.

Analysis of the accuracy of orientation (κ) was performed using the directional statistics software module *DirStat* [89], embedded into the R programming environment (http://www.r-project.org). We used the *acorn* function to estimate the accuracy of orientation κ, and the *vmtests.on.files* function to statistically test equality of κ between strains.

## Supporting Information

Table S1 **Random motility by vegetative cells.** A. Speeds. Speeds (V) are shown as means ± s.d. for 3 independent experiments for each strain. Negative copy numbers refer to antisense inhibition constructs. B. Pairwise statistical tests. Pair-wise statistical comparison between average values of speed V between five strains, two-tailed *t*-test, based on total N (number of cells per strain). Statistically significant comparisons are highlighted by yellow shaded boxes.
(DOC)

Table S2 **Chemotaxis by aggregation-competent cells.** A. Speeds and accuracies of chemotaxis. Traditional 90% confidence intervals for κ are designated in brackets. Speeds are shown as means ± s.d. V - average speed of migration in µm/min. κ - accuracy of orientation. N - number of cells tracked. n - number of movement steps. B. Pairwise statistical tests. Pairwise comparisons were made of the accuracy of orientation (κ) and speed (V) amongst three strains. Two sample tests based on the von Mises distribution and two-tailed *t*-tests were used for κ and V respectively. Statistically significant comparisons are highlighted by yellow shaded boxes.
(DOC)
