# Supplementary material for: Dictyostelium discoideum Nucleoside Diphosphate Kinase C Plays a Negative Regulatory Role in Phagocytosis, Macropinocytosis and Exocytosis
Source: PLoS One. 2011 Oct 4;6(10):e26024. doi: 10.1371/journal.pone.0026024 (PMC3186806; doi:10.1371/journal.pone.0026024)
Supplement: Table S1 — Random motility by vegetative cells. A. Speeds. Speeds (V) are shown as means ± s.d. for 3 independent experiments for each strain. Negative copy numbers refer to antisense inhibition constructs. B. Pairwise statistical tests. Pair-wise statistical comparison between average values of speed V between five strains, two-tailed t-test, based on total N (number of cells per strain). Statistically significant comparisons are highlighted by yellow shaded boxes. (DOC) [file pone.0026024.s001.doc]

**Table S1. Random motility by vegetative cells.**

1. **Speeds**

|  | **ANTISENSE STRAINS**  **(Construct copy number)** | | **OVEREXPRESSOR STRAINS**  **(Construct copy number)** | | **WILD TYPE** |
| --- | --- | --- | --- | --- | --- |
|  | 500#10  (-437) | 500#19  (-239) | 520#16  (+123) | 520#26  (+144) | AX2 |
| V | 4.4 ± 1.0  (N=23) | 3.7 ± 0.6  (N=21) | 3.5 ± 0.8  (N=14) | 4.1 ± 0.9  (N=17) | 2.4 ± 0.8  (N=19) |
|  | 3.9 ± 1.0  (N=17) | 3.3 ± 0.6  (N=14) | 3.6 ± 0.7  (N=21) | 4.2 ± 1.0  (N=19) | 2.4 ± 1.4 (N=17) |
|  | 4.0 ± 1.1  (N=11) | 3.7 ± 0.8  (N=19) | 3.4 ± 0.7  (N=21) | 4.2 ± 1.0  (N=20) | 3.0 ± 1.1  (N=20) |
| Pooled data: | | | | | |
| N | 51 | 54 | 56 | 56 | 56 |
| n | 8677 | 9262 | 9727 | 9602 | 9558 |
| V | **4.2 ± 1.0** | **3.6 ± 0.7** | **3.5 ± 0.7** | **4.2 ± 1.0** | **2.6 ± 1.1** |

1. **Pairwise statistical tests**

|  | 500#10 | 500#19 | AX2 | 520#16 | 520#26 |
| --- | --- | --- | --- | --- | --- |
| 500#10 |  | p(V) < 10^-3^ | p(V) < 10^-9^ | p(V) < 10^-4^ | p(V) > 0.9 |
| 500#19 |  |  | p(V) < 10^-7^ | p(V) ≈ 0.45 | p(V) < 10^-3^ |
| AX2 |  |  |  | p(V) ≈ 10^-6^ | p(V) < 10^-9^ |
| 520#16 |  |  |  |  | p(V) < 10^-4^ |
| 520#26 |  |  |  |  |  |

.
