# Supplementary material for: Dictyostelium discoideum Nucleoside Diphosphate Kinase C Plays a Negative Regulatory Role in Phagocytosis, Macropinocytosis and Exocytosis
Source: PLoS One. 2011 Oct 4;6(10):e26024. doi: 10.1371/journal.pone.0026024 (PMC3186806; doi:10.1371/journal.pone.0026024)
Supplement: Table S2 — Chemotaxis by aggregation-competent cells. A. Speeds and accuracies of chemotaxis. Traditional 90% confidence intervals for κ are designated in brackets. Speeds are shown as means ± s.d. V - average speed of migration in µm/min. κ - accuracy of orientation. N - number of cells tracked. n - number of movement steps. B. Pairwise statistical tests. Pairwise comparisons were made of the accuracy of orientation (κ) and speed (V) amongst three strains. Two sample tests based on the von Mises distribution and two-tailed t-tests were used for κ and V respectively. Statistically significant comparisons are highlighted by yellow shaded boxes. (DOC) [file pone.0026024.s002.doc]

**Table S2. Chemotaxis by aggregation-competent cells.**

1. **Speed and accuracy of chemotaxis.**

|  | **ANTISENSE STRAINS**  **(Construct copy number)** | | **OVEREXPRESSOR STRAINS**  **(Construct copy number)** | | **WILD TYPE** |
| --- | --- | --- | --- | --- | --- |
|  | 500#10  (-437) | 500#19  (-239) | 520#16  (+123) | 520#26  (+144) | AX2 |
| κ | **2.39 (2.24, 2.46)** | **1.95 (1.88, 2.05)** | **1.94 (1.87, 2.06)** | **2.49 (2.32, 2.58)** | **2.76 (2.53, 2.92)** |
| V | **13.9 ± 2.7** | **14.2 ± 2.2** | **15.5 ± 2.8** | **15.8 ± 1.9** | **13.2 ± 1.9** |
| N | 32 | 39 | 33 | 45 | 26 |
| n | 1895 | 1695 | 1517 | 1531 | 802 |

1. **Pairwise statistical tests.**

|  | 500#10 | 500#19 | AX2 | 520#16 | 520#26 |
| --- | --- | --- | --- | --- | --- |
| 500#10 |  | p(V) ≈ 0.6 | p(V) > 0.25 | p(V) ≈ 0.02 | p(V) < 10^-3^ |
| 500#19 | p(κ) < 10^-6^ |  | p(V) ≈ 0.06 | p(V) ≈ 0.03 | p(V) < 10^-3^ |
| AX2 | p(κ) ≈ 0.003 | p(κ) < 10^-9^ |  | p(V) < 10^-3^ | p(V) < 10^-3^ |
| 520#16 | p(κ) < 10^-6^ | p(κ) ≈ 0.47 | p(κ) < 10^-9^ |  | p(V) ≈ 0.6 |
| 520#26 | p(κ) ≈ 0.03 | p(κ) < 10^-7^ | p(κ) ≈ 0.03 | p(κ) < 10^-7^ |  |
